# Uric acid formation is driven by crosstalk between skeletal muscle and other cell types

**DOI:** 10.1172/jci.insight.171815

**Published:** 2024-01-23

**Authors:** Spencer G. Miller, Catalina Matias, Paul S. Hafen, Andrew S. Law, Carol A. Witczak, Jeffrey J. Brault

**Affiliations:** 1 Indiana Center for Musculoskeletal Health and; 2 Department of Anatomy, Cell Biology & Physiology, Indiana University School of Medicine, Indianapolis, Indiana, USA.; 3 Department of Kinesiology, East Carolina University, Greenville, North Carolina, USA.

**Keywords:** Metabolism, Muscle Biology, Bioenergetics, Skeletal muscle

## Abstract

Hyperuricemia is implicated in numerous pathologies, but the mechanisms underlying uric acid production are poorly understood. Using a combination of mouse studies, cell culture studies, and human serum samples, we sought to determine the cellular source of uric acid. In mice, fasting and glucocorticoid treatment increased serum uric acid and uric acid release from ex vivo–incubated skeletal muscle. In vitro, glucocorticoids and the transcription factor FoxO3 increased purine nucleotide degradation and purine release from differentiated muscle cells, which coincided with the transcriptional upregulation of AMP deaminase 3, a rate-limiting enzyme in adenine nucleotide degradation. Heavy isotope tracing during coculture experiments revealed that oxidation of muscle purines to uric acid required their transfer from muscle cells to a cell type that expresses xanthine oxidoreductase, such as endothelial cells. Last, in healthy women, matched for age and body composition, serum uric acid was greater in individuals scoring below average on standard physical function assessments. Together, these studies reveal skeletal muscle purine degradation is an underlying driver of uric acid production, with the final step of uric acid production occurring primarily in a nonmuscle cell type. This suggests that skeletal muscle fiber purine degradation may represent a therapeutic target to reduce serum uric acid and treat numerous pathologies.

## Abstract 2

<p>During atrophic conditions, skeletal muscle degrades endogenous purines that are released and converted to uric acid by other cell types, such as endothelial cells.</p>

## Graphical abstract

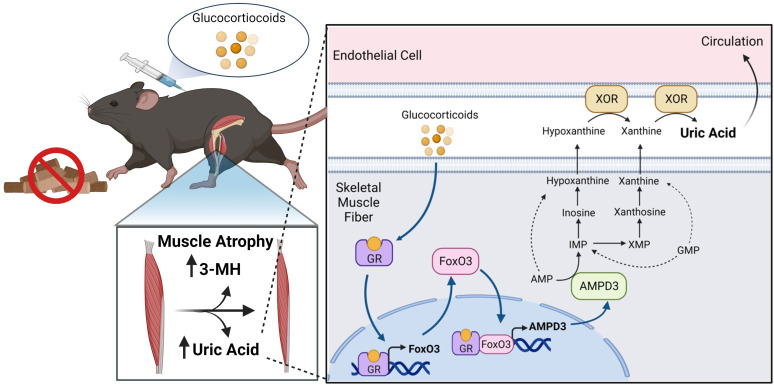

## Introduction

Increased serum uric acid is an independent risk factor for the development and poor prognosis of many pathologies, including cancer (1), cardiovascular disease (2, 3), chronic kidney disease (4), type 2 diabetes (5), and liver disease (6). It is also positively associated with disease risk factors, such as components of metabolic syndrome (7) and inflammation (8). The production of uric acid probably plays a direct role in the pathology since inhibition of xanthine oxidase (the enzyme that synthesizes uric acid) has been shown to alleviate contributing disorders such as endothelial dysfunction (9), insulin resistance (10), hepatic steatosis (11), and muscle atrophy (12–15). Surprisingly, the mechanisms underlying increased uric acid production and its cellular source are poorly understood.

Uric acid is produced exclusively by the enzyme xanthine oxidoreductase (XOR), which catalyzes the irreversible oxidation of hypoxanthine and xanthine in the final step of purine degradation in humans. Hypoxanthine and xanthine are produced from the breakdown of purine nucleotide monophosphates (AMP, GMP, IMP, and XMP), which are generated from the catabolism of ATP/GTP, DNA/RNA, or de novo purine nucleotide synthesis. XOR is heterogeneously expressed in mammalian tissues, being most abundant in vascular endothelial cells, intestinal epithelial cells, hepatocytes, and adipocytes (16–19). In cell types that do not express XOR, such as skeletal muscle fibers (16, 19), purine nucleotide degradation is presumed to culminate in release of hypoxanthine and xanthine, which may undergo oxidation to uric acid in a XOR-expressing cell type or by circulating XOR (20, 21). Thus, because XOR-expressing cells can oxidize their own purines and those released by non-XOR-expressing cells, the predominant cellular source(s) supplying purine precursors for uric acid formation remain unknown.

Several lines of evidence implicate skeletal muscle fibers as a major source of purine precursors used for uric acid formation during muscle wasting conditions. Diseases and conditions associated with increased rates of muscle protein degradation and atrophy are also characterized by reduced muscle ATP and total adenine nucleotides and increased serum uric acid (22). Rodent studies have identified the metabolic enzyme AMP deaminase 3 (AMPD3; AMP→IMP+NH_3_), which is sufficient to increase adenine nucleotide degradation (23, 24), as one of the most highly upregulated genes in muscles atrophying in response to diseases (25) and disuse/unloading (26). However, whether purine nucleotide degradation and release are increased in skeletal muscles during conditions associated with atrophy, and whether muscle purine release would be sufficient to drive uric acid formation in a XOR-expressing cell type, are unknown.

Therefore, the goals of this study were to determine if purine nucleotide degradation is increased in skeletal muscle with heightened rates of protein degradation (i.e., in the process of atrophying) and, if so, to determine if increased muscle purine efflux is sufficient to stimulate uric acid formation. To address these goals, we analyzed serum uric acid and purine release from isolated mouse muscles and myotubes during fasting-, glucocorticoid-, and FoxO-induced atrophy; performed in vitro coculture experiments to trace the fate of purines released by atrophying myotubes; and compared serum uric acid and markers of protein degradation in humans with different muscle performance levels.

## Results

### Skeletal muscle uric acid efflux is increased after fasting and dexamethasone treatment.

**Figure 1:**
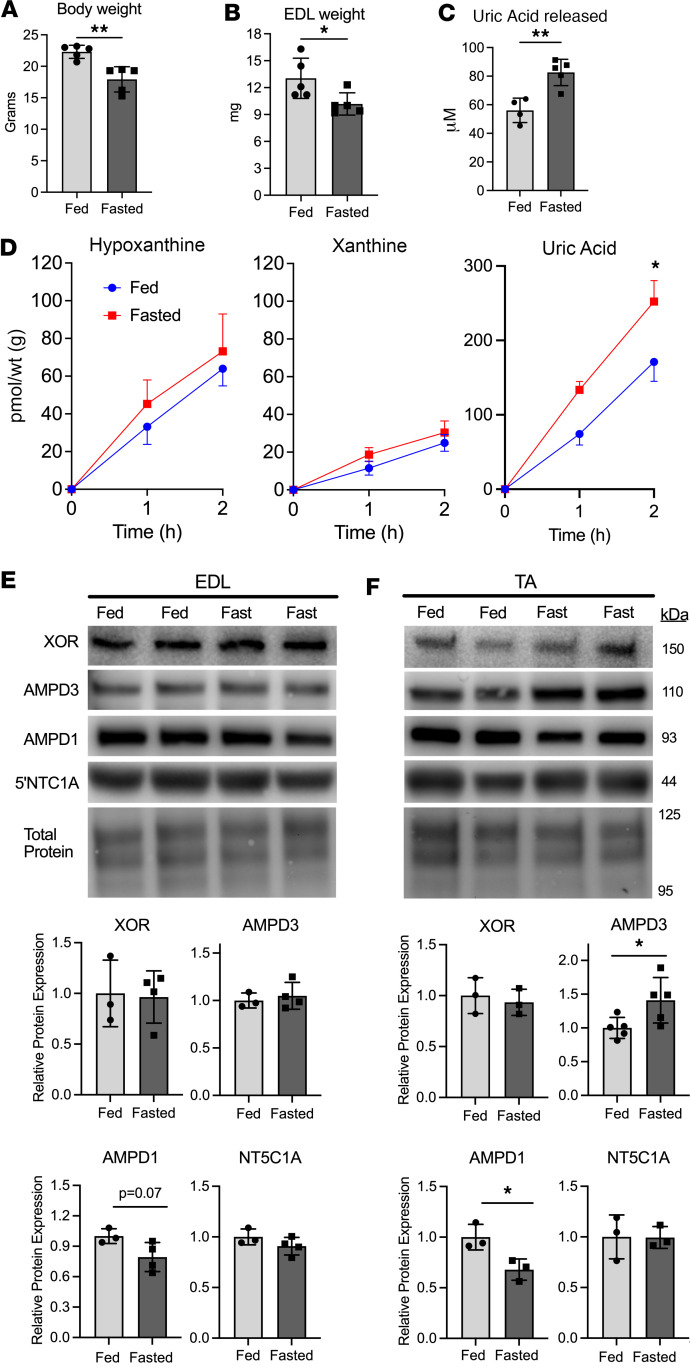
Fasting causes increased uric acid release from skeletal muscle. Female C57BL/6J mice had food removed for 48 hours. (**A**) Body weight. (**B**) EDL muscle wet weights. (**C**) Uric acid concentration in serum collected at sacrifice. (**D**) Concentrations of the purine nucleotide breakdown products hypoxanthine, xanthine, and uric acid released from EDL muscles during ex vivo incubation. *n* = 4–5/group, 2-way ANOVA, Holm-Šídák multiple comparisons test. *=*P* < 0.001 (**D**). Protein expression levels of the purine nucleotide-degrading enzymes xanthine oxidoreductase (XOR), AMP deaminases 1/3 (AMPD1/3), and cytosolic 5′nucleotidase 1 (NT5C1A), from (**E**) EDL or (**F**) tibialis anterior (TA) muscle. Panels **A**–**C**, **E**, and **F**: 2-tailed unpaired *t* test, *=*P* < 0.05, **=*P* < 0.01.

Increased serum uric acid levels are common among diseases and conditions in which muscle atrophy is prevalent (22). To determine if atrophying skeletal muscles have increased purine nucleotide degradation, we measured release of purine nucleotide degradation products (hypoxanthine, xanthine, uric acid) from incubated mouse extensor digitorum longus (EDL) muscles after fasting, a well-characterized model of accelerated protein degradation and atrophy (27–29). Fasting for 48 hours reduced body and EDL weight (Figure 1, A and B) and increased serum uric acid (Figure 1C). Incubated EDL muscles from fasted mice produced greater uric acid compared with nonfasted controls (Figure 1D). No differences were detected in release of hypoxanthine or xanthine. Further, no differences in protein expression of the adenine nucleotide-degrading enzymes AMPD1, AMPD3, cytosolic 5′nucleotidase 1 (NT5C1A), or XOR were detected in the EDL muscles (Figure 1E). Notably, we did detect an increase in AMPD3 protein levels in fasted tibialis anterior (TA) muscles (Figure 1F). Unfortunately, TA muscles are too large to allow sufficient oxygen perfusion during ex vivo incubations (30); hence, purine efflux measures as done here are not feasible in TA muscles.

**Figure 2:**
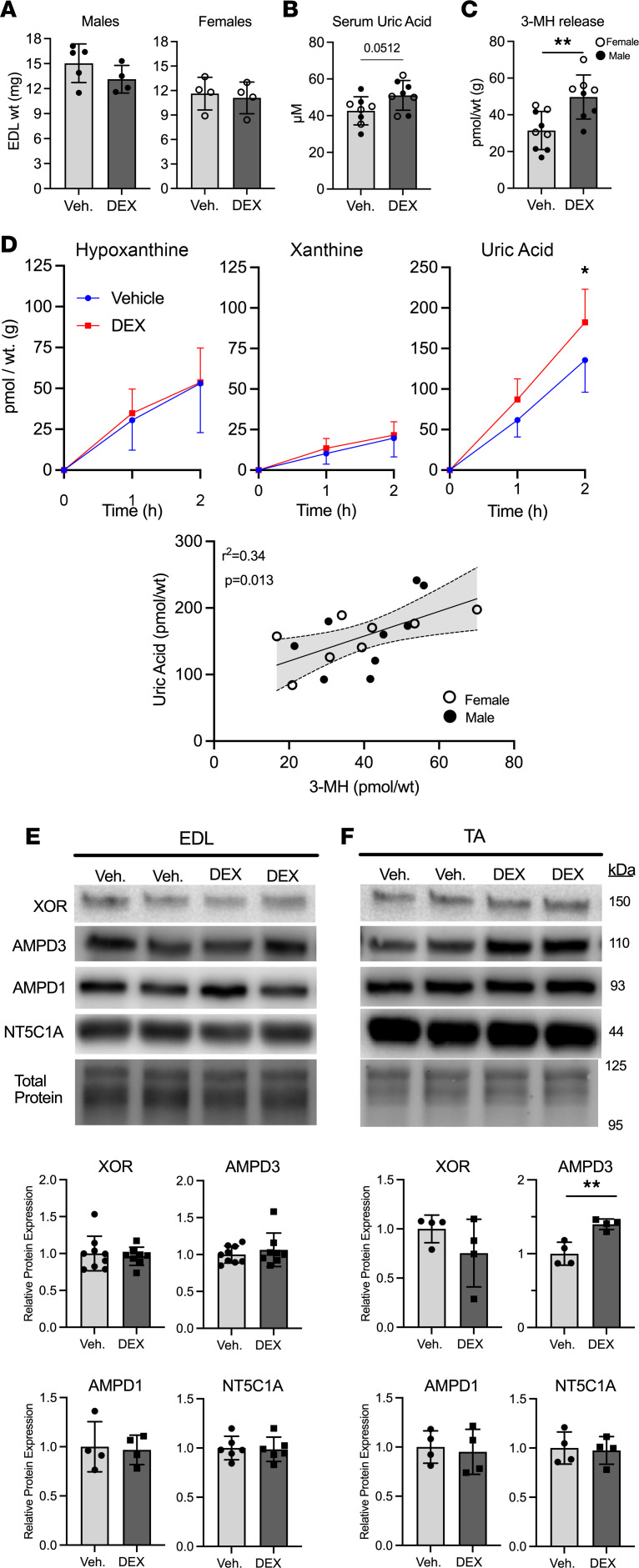
Glucocorticoid treatment is sufficient to increase uric acid release from skeletal muscle. Male and female C57BL/6J mice were treated with dexamethasone (DEX; 5 mg/kg) or vehicle (Veh.) for 5 days. (**A**) Male and female EDL muscle wet weights after treatment period. (**B**) Uric acid concentration in serum collected at sacrifice. (**C**) Concentration of 3-methylhistidine (3-MH) measured in EDL incubation buffer after 2 hours. (**D**) Concentrations of the purine nucleotide breakdown products hypoxanthine, xanthine, and uric acid released from EDL muscles during ex vivo incubation. *n* = 8/9 group combination of male and female, 2-way ANOVA, Holm-Šídák multiple comparisons. *=*P* < 0.05. Linear correlation, including 95% confidence intervals, between uric acid and 3-MH release from EDL muscles after 2 hours’ incubation. Protein expression levels of xanthine oxidoreductase (XOR), AMP deaminases 1/3 (AMPD1/3), and cytosolic 5′nucleotidase 1 (NT5C1A), from (**E**) EDL or (**F**) tibialis anterior (TA) muscle. Panels **A**–**C**, **E**, and **F**: 2-tailed unpaired *t* test, **=*P* < 0.01.

Increased muscle protein degradation in response to fasting is partly mediated by increased circulating glucocorticoids (31). To determine if increased glucocorticoid exposure is sufficient to increase muscle purine efflux, mice were injected with the synthetic glucocorticoid, dexamethasone (DEX), daily for 5 days. DEX treatment did not cause a significant reduction in EDL muscle weights in male or female mice (Figure 2A) but did cause a 17% increase (*P* = 0.051) in serum uric acid (Figure 2B). Additionally, DEX increased 3-MH release from incubated EDL muscles (Figure 2C), indicating that myofibrillar protein degradation rate was increased (32, 33). Incubated EDL muscles from DEX-treated mice also released more uric acid compared with vehicle treated (Figure 2D), leading to a positive correlation between uric acid and 3-MH release (Figure 2D). Importantly, no differences were found between serum uric acid or release of 3-MH, hypoxanthine, xanthine, or uric acid between males and females (Supplemental Figure 1; supplemental material available online with this article; https://doi.org/10.1172/jci.insight.171815DS1). Therefore, the sexes were combined for the above analysis. No differences in protein expression of purine nucleotide-degrading enzymes were detected in DEX-treated EDL muscles (Figure 2E). However, similar to fasted mice, an increase in AMPD3 protein was observed in DEX-treated TA muscles (Figure 2F).

### Purine nucleotide degradation and purine efflux are increased in atrophying muscle cells.

**Figure 3:**
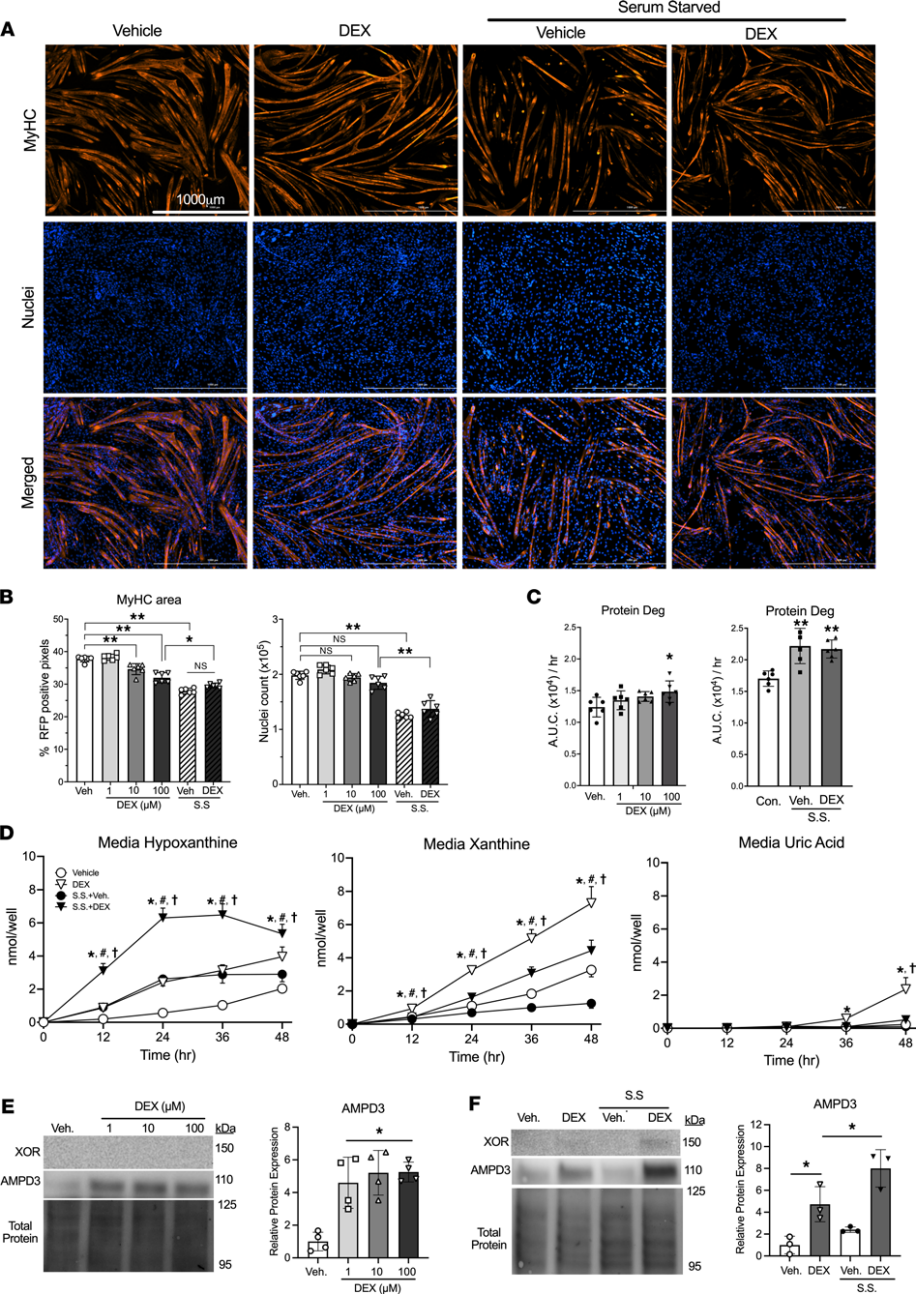
Purine nucleotide degradation is increased in atrophying myotubes but culminates in the release of hypoxanthine and xanthine due to their lack of XOR expression. C2C12 myotubes were treated with dexamethasone (DEX) and/or serum starvation (S.S.) treatments for 48 hours. (**A**) Representative immunofluorescence images after staining for myosin heavy chain (MyHC) and nuclei (DAPI) in vehicle, 100 μM DEX, S.S.+Veh., or S.S.+100 μM DEX. (**B**) Quantifications of MyHC area and nuclei count per well. *n* = 6 wells/condition and 100 images/well, *=*P* < 0.05, **=*P* < 0.01, 1-way ANOVA with Tukey’s multiple comparisons. (**C**) Protein degradation rates were determined as media accumulation of ^13^C_9_^15^N-phenylalanine 6–24 hours after dexamethasone treatment or after S.S.+Veh., S.S.+100 μM DEX, or untreated controls. *n* = 6 wells/condition, *=*P* < 0.05, **=*P* < 0.01 vs. Veh/control, 1-way ANOVA with Dunnett’s multiple comparisons. (**D**) Media concentrations of the purine nucleotide breakdown products hypoxanthine, xanthine, and uric acid during 100 μM DEX and/or S.S. treatment. *=*P* < 0.05 DEX vs. Veh., ^#^=*P* < 0.05 S.S.+Veh. vs. Veh., ^†^=*P* < 0.05 S.S.+Veh. vs. S.S.+DEX 100 μM. *n* = 6 wells/condition, 2-way ANOVA with Tukey’s multiple comparisons. (**E** and **F**) AMPD3 protein expression after 48 hours of DEX and/or S.S. treatments. *=*P* < 0.05 vs. Veh. or indicated group.

Muscle tissue comprises numerous cell types, such as muscle fibers, endothelial cells, macrophages, fibro-adipogenic progenitor cells, and satellite cells. Immunohistochemical staining of muscle has shown XOR expression is overwhelmingly localized to vascular cells (16, 17). To determine if purine nucleotide degradation is specifically upregulated in muscle cells during atrophy, purine release was measured from C2C12 myotubes during DEX and/or serum starvation (S.S.) treatments. After 48 hours, myotube atrophy was confirmed by quantifying myosin heavy chain (MyHC) area (Figure 3A). Compared with vehicle, myotubes treated with 10 or 100 μM DEX had less MyHC area but no differences in nuclei count (Figure 3B). S.S. myotubes had the lowest MyHC area and fewest nuclei (Figure 3B). The reduction in MyHC area was preceded by increases in protein degradation rates between 6 and 24 hours after DEX and/or S.S. treatment (Figure 3C).

After verifying these treatments cause atrophy, we determined if purine nucleotide degradation was increased by measuring hypoxanthine, xanthine, and uric acid in the media. Treatment with 100 mm DEX caused a significant increase in hypoxanthine and xanthine release by 12 hours, eventually reaching peak levels at 48 hours that were 3.6- and 1.8-fold greater than vehicle, respectively (Figure 3D). The increase was similar among the different DEX doses tested (Supplemental Figure 2). S.S.+Veh also caused an increase in hypoxanthine release, which peaked at 24 hours and then remained unchanged (Figure 3D). S.S.+DEX resulted in the greatest hypoxanthine release among groups, and similar to S.S.+Veh, remained unchanged after peaking at 24 hours (Figure 3D). Interestingly, S.S. negatively affected xanthine release, with S.S.+Veh groups releasing less xanthine than Veh treated, and S.S.+DEX groups releasing more than S.S.+Veh and Veh, but less than DEX (Figure 3D). Importantly, unlike its precursors hypoxanthine and xanthine, uric acid was undetected in all groups until 36 hours, when it was detected in DEX groups at a miniscule 0.4 nmol/well (Figure 3D). By 48 hours it had risen to 2 nmol/well in the DEX group and 0.5 nmol/well in the S.S.+DEX group, respectively (Figure 3D). These increases in purine efflux in response to DEX coincided with 4-fold increased AMPD3 protein expression (Figure 3, E and F). In contrast, XOR was undetectable except for a faint, but unquantifiable, band in the S.S.+DEX condition (Figure 3F).

### Increased FoxO3 activity is sufficient to increase purine nucleotide degradation and AMPD3 protein expression in myotubes.

**Figure 4:**
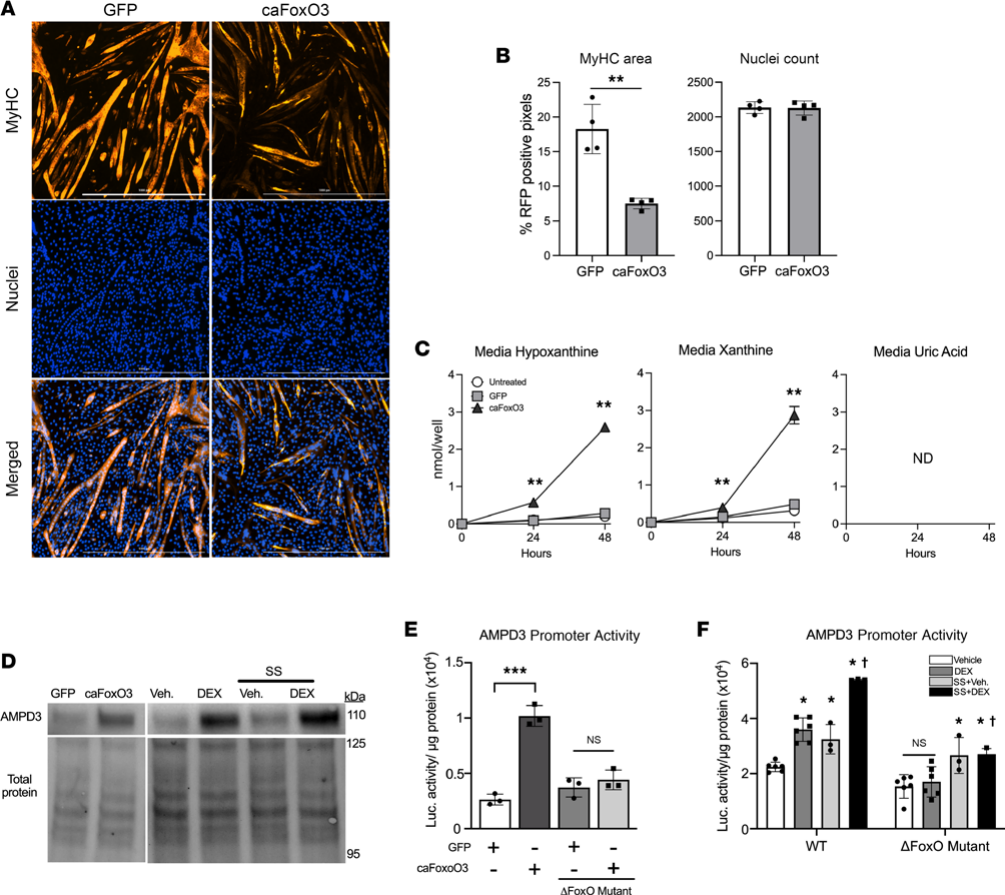
Increased FoxO3 activity is sufficient to induce myotube purine nucleotide degradation and is required for DEX upregulation of AMPD3 promoter activity. C2C12 myotubes were allowed to differentiate for 4 days, then infected with adenovirus encoding a constitutively active FoxO3 isoform (caFoxO3) or GFP for 48 hours. (**A**) Representative immunofluorescence images after staining for MyHC and nuclei (DAPI). Scale bar, 1,000 μm. (**B**) Quantification of MyHC area and nuclei count per well. *n* = 4 wells/group and 6 random images/well. **=*P* < 0.01. (**C**) Media concentrations of the purine nucleotide breakdown products hypoxanthine, xanthine, and uric acid. **=*P* < 0.01 vs. GFP. ND, not detected. (**D**) Western blots for AMPD3 at 24 hours after GFP and caFoxO3 infection or DEX and S.S. treatments. (**E** and **F**) Prior to differentiation, C2C12 myoblasts were transfected with luciferase reporter plasmids containing 1 Kb of the AMPD3 proximal promoter region, with or without substitution mutations in the consensus FoxO binding site (ΔFoxO Mutant). (**E**) AMPD3 promoter activity measured 24 hours after GFP or caFoxO3 infection. One-way ANOVA, Tukey’s multiple comparisons. ***=*P* < 0.0001. (**F**) AMPD3 promoter activity after 24 hours’ treatment with 100 μM DEX and/or S.S. *=*P* < 0.05 vs. Veh, ^†^=*P* < 0.05 S.S.+DEX vs. DEX. Two-way ANOVA, Tukey’s multiple comparisons.

Glucocorticoids and fasting induce protein degradation and muscle atrophy, in part, by increasing the activity of FoxO transcription factors (28, 29, 34). Therefore, to determine if increased FoxO activity is sufficient to stimulate muscle purine nucleotide degradation, we infected C2C12 myotubes with an adenovirus encoding a constitutively active FoxO3 (caFoxO3) (28, 35). As expected, myotubes expressing caFoxO3 had less MyHC area but similar nuclei count compared to GFP controls (Figure 4, A and B). Moreover, caFoxO3 increased hypoxanthine and xanthine release, while uric acid was undetected (Figure 4C), demonstrating that increased FoxO activity is sufficient to stimulate purine nucleotide degradation in muscle. As with DEX treatment, the elevated production of hypoxanthine and xanthine with caFoxO3 expression coincided with demonstrable upregulation of AMPD3 (Figure 4D).

The AMPD3 promoter region contains a consensus FoxO binding site 124 bases upstream of the transcriptional start site. To determine if increased AMPD3 protein is related to FoxO-dependent activation of its promoter region, myoblasts were transfected with custom luciferase reporter plasmids containing 1.1 kb of the mouse AMPD3 proximal promoter region, with or without a mutation in the FoxO binding site (ΔFoxO mutant). CaFoxO3 expression was sufficient to increase AMPD3 promoter activity by 3.9-fold, which was prevented by mutating its binding site in the AMPD3 promoter region (Figure 4E). Moreover, AMPD3 promoter activity was increased by DEX and S.S. treatment by a FoxO-dependent mechanism (Figure 4F).

### Purines released from atrophying myotubes drive uric acid formation by XOR-expressing cells.

**Figure 5:**
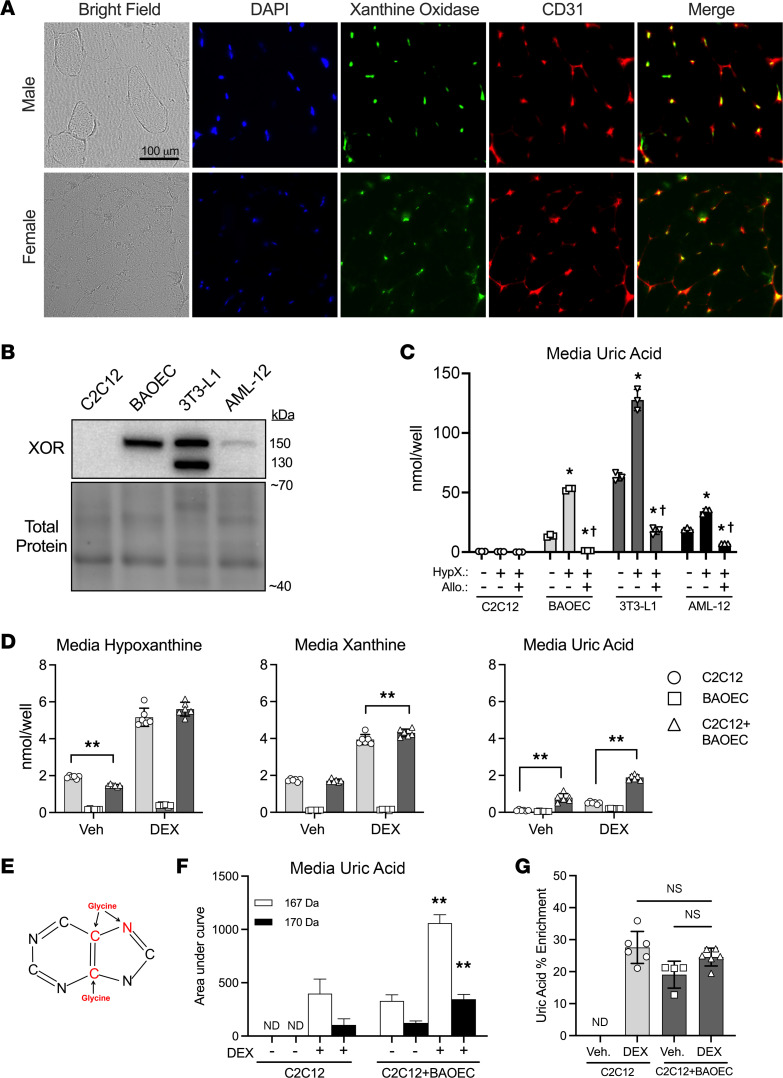
XOR-expressing cells oxidize exogenous purines released by muscle. (**A**) Mouse tibialis anterior cross sections were visualized by bright-field, stained for nuclei (DAPI), or immunofluorescence stained for the endothelial cell marker CD31 and xanthine oxidase. Merge is the combination of CD31 and xanthine oxidase images. (**B** and **C**) Myotubes (C2C12), aortic endothelial cells (BAOECs), adipocytes (3T3-L1), and hepatocytes (AML-12) were incubated with vehicle, 50 μM hypoxanthine, or 50 μM hypoxanthine + 100 μM allopurinol (XOR inhibitor) for 48 hours. Media samples were collected over time, and purines were measured by UPLC. Cells were harvested for protein extraction and XOR expression. (**B**) Western blots for XOR. (**C**) Media uric acid concentration after 48 hours’ treatment. One-way ANOVA within each cell type. *=*P* < 0.05 vs. untreated condition. ^†^=*P* < 0.05 vs. hypoxanthine-treated condition. (**D**–**G**) C2C12 myotubes were grown in medium supplemented with ^13^C^15^N-glycine to label purines. At day 5 of differentiation, ^13^C^15^N-glycine–containing medium was replaced by normal medium–containing vehicle or 100 μM DEX, then given to wells with C2C12 myotubes only, BAOECs only, or C2C12+BAOEC cocultures. (**D**) Media purine concentrations after 48 hours. **=*P* < 0.01, 2-way ANOVA, Tukey’s multiple comparisons test. (**E**) Uric acid illustration with atoms donated from glycine highlighted in red. (**F**) Media levels of normal (167 Da) vs. heavy-labeled (170 Da) uric acid from the same samples as **C**. Two-way ANOVA. **=*P* < 0.01 vs. C2C12 DEX. (**G**) Percentage of heavy-labeled uric acid per total uric acid measured. One-way ANOVA, Tukey’s multiple comparisons. ND, not detected.

Since whole muscle tissues release uric acid (Figures 1 and 2), but uric acid production by muscle cells is extremely slow (Figures 3 and 4), we next sought to verify that xanthine oxidase is expressed by some muscle-resident cell types, but not muscle fibers, in whole muscle. Immunofluorescence staining for xanthine oxidase was performed on cross sections of TA muscles of male and female mice. As expected, XOR was not detectable in the muscle fibers but was detectable in numerous other cells, many of which expressed the endothelial cell marker CD31 (Figure 5A). Importantly, not all XOR-positive cells were CD31 positive, suggesting that endothelial cells are not the exclusive source of XOR in whole skeletal muscle.

To determine if myotube purines could be oxidized to uric acid by XOR-expressing cell types, first, XOR protein expression was confirmed in bovine aortic endothelial cells (BAOECs) (36), 3T3-L1 adipocytes (18), and AML-12 hepatocytes (18) by Western blot (Figure 5B). Next, we tested whether these cells could oxidize physiological levels of exogenous purines. All 4 cell types (C2C12, BAOEC, 3T3-L1, and AML-12) consumed hypoxanthine, as demonstrated by progressive reductions over time in hypoxanthine media concentrations (Supplemental Figure 3, A, D, G, and J). However, only XOR-expressing cells consumed xanthine (Supplemental Figure 3, B, E, H, and K). In cell types expressing XOR, hypoxanthine and xanthine both caused dramatic increases in uric acid production, which was prevented by the XOR inhibitor allopurinol (Figure 5C and Supplemental Figure 3, C, F, I, and L). We then tested whether coculturing myotubes with BAOECs would enable the oxidation of purines released from myotubes and whether a myotube atrophy stimulus would augment uric acid formation. Prior to coculturing, myotube purines were labeled with ^13^C^15^N-glycine, which supplies 2 carbons and 1 nitrogen atom to the purine ring during de novo purine synthesis (Figure 5E). At the start of the coculture, ^13^C^15^N-glycine medium was replaced with nonlabeled medium containing vehicle or DEX, then added to wells containing C2C12 only, BAOEC only, or C2C12+BAOEC. As before in C2C12 cells only (Figure 3D), DEX increased hypoxanthine, xanthine, and uric acid in the media (Figure 5D). However, the coculture of C2C12+BAOEC led to 3.5- and 9.5-fold greater uric acid in C2C12+BAOEC versus C2C12 or BAOEC only (Figure 5D). In addition, heavy-labeled uric acid (170 Da) was only detected in wells containing myotubes and was 2.8-fold greater in C2C12+BAOEC than C2C12 after DEX (Figure 5F). Despite the substantial increase in uric acid production in C2C12+BAOEC cocultures, the percentage of heavy-labeled uric acid per total uric acid was similar between C2C12 and C2C12+BAOEC after DEX (Figure 5G). This indicates that the increased uric acid produced in the cocultures is derived solely from the myotube purines, not from greater purine degradation in the BAOECs.

### Serum uric acid is greater in individuals with low physical performance.

**Figure 6:**
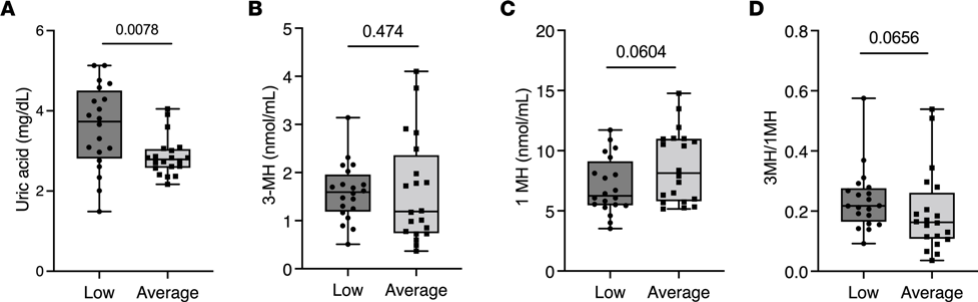
Serum uric acid is greater in individuals with below-average physical fitness performance. Serum was collected from adult women with either low or average physical performance. (**A**) Serum uric acid levels. (**B**) Serum 3-MH. (**C**) Serum 1-MH. (**D**) Ratio of 3-MH to 1-MH. Box plots show the interquartile range (box), median (line), and minimum and maximum (whiskers). *n* = 20/group, unpaired 2-tailed *t* test.

Deficits in contractile performance are known to precede muscle mass loss during aging and atrophy-inducing conditions (37–39). We showed earlier that muscle purine release was elevated prior to increased protein degradation and myotube atrophy and was equally increased in myotubes given DEX doses insufficient to increase protein degradation and cause atrophy (Figure 3 and Supplemental Figure 2). Based on these findings, we questioned whether serum uric acid levels would be higher in individuals with lower muscle performance. Since serum uric acid increases with lean body mass and may differ by age (40, 41), female participants were matched for age, height, weight, body mass index (BMI), and estimated lean body mass (appendicular mass/height^2^) (Table 1). Low performers had lower max grip strength, longer time to complete 5 sit-to-stands, fewer sit-to-stands completed in 30 seconds, and a trend (*P* = 0.06) for shorter 6-minute walking distance (Table 1). Serum uric acid levels were significantly greater in low (3.59 ± 1.04 mg/dL) versus average (2.86 ± 0.49 mg/dL) performance groups (Figure 6), but no differences in serum creatinine or creatine kinase activity (markers of kidney function, muscle mass, and muscle damage) were detected (Table 1).

Serum 3-MH, an amino acid found predominantly in myofibrillar actin and myosin (32), can be used as a marker of muscle protein degradation. However, serum 3-MH is also influenced by dietary meat intake (32). Therefore, the ratio of 3-MH to 1-MH (which is uniquely found in nonhuman animal muscle; ref. 32) can be used to more accurately estimate muscle protein degradation during nonfasted states (42). Serum 3-MH/1-MH trended higher in the low-performance group (*P* = 0.06, Figure 6), suggesting accelerated muscle protein degradation in the low performers.

## Discussion

The present study sought to determine if purine nucleotide degradation and purine release are increased in skeletal muscles undergoing atrophy and whether elevated muscle purine release could stimulate uric acid synthesis. In mice, fasting- and glucocorticoid-induced protein degradation coincided with increased serum uric acid and uric acid efflux from isolated EDL muscles. In cultured myotubes, glucocorticoid- and caFoxO3-induced treatment coincided with increased hypoxanthine and xanthine efflux but little to no uric acid production, which is consistent with poor XOR expression in muscle cells. Coculturing myotubes with BAOECs, which do express XOR, enabled the oxidation of myotube-released hypoxanthine and xanthine to uric acid. Last, in humans, serum uric acid was greater in individuals with below-average physical performance who also had a trend for increased serum 3-MH/1-MH ratio, an indicator of increased muscle protein degradation. Thus, our findings demonstrate that purine nucleotide degradation and purine release are increased in muscle cells undergoing atrophy, and muscle purine release stimulates uric acid synthesis by XOR-expressing cells, such as endothelial cells. Given that skeletal muscles are the most abundant bodily tissue, contain the greatest concentrations of adenine nucleotides (43), and have enzymatic capacity for de novo purine synthesis (44), our data suggest that skeletal muscle is a major precursor source of circulating uric acid.

Our incubated mouse muscle experiments agree with studies of exercise in humans demonstrating that whole muscles can degrade purines and release uric acid into the circulation (20, 21). However, our series of subsequent in vitro experiments extend these findings to explore the role of different cell types. First, we found that induction of protein degradation and/or atrophy of multinucleated muscle cells (C2C12 myotubes) resulted in increased purine nucleotide degradation, which culminated predominantly in the release of hypoxanthine and xanthine, not uric acid. Further, XOR-expressing cells readily converted exogenous hypoxanthine and xanthine to uric acid, which was prevented by the XOR inhibitor allopurinol. Last, coculturing myotubes with BAOECs (which express XOR) enabled the oxidation of myotube-derived purines to uric acid, which was enhanced when the myotubes were undergoing atrophy. Therefore, the observed release of uric acid from whole muscles probably involves transfer of purines from muscle cells to endothelial cells or other cell types in whole muscle that express XOR, such as adipocytes, fibro-adipogenic progenitors, or immune cells.

Interestingly, the atrophying EDL muscles and myotubes had increased purine nucleotide degradation despite ample access to exogenous nutrients and oxygen, without any contractile demand. Increased skeletal muscle purine nucleotide degradation and purine efflux readily occur during hypoxia and intense contractions (21, 45), during which ATP synthesis is unable to match ATP hydrolysis. This mismatch leads to increased activation of AMPD and, thus, degradation of nucleotides to maintain the cellular energy state (20, 21, 45). Therefore, our findings may reflect the ability of fasting, glucocorticoids, and FoxO to impair muscle bioenergetic function to an extent that resting ATP/ADP could not be maintained. This possibility is supported by NMR measurements of decreased phosphocreatine and calculated free energy of ATP hydrolysis (ΔG_ATP_) in rat gastrocnemius muscle after DEX treatment (46). This would be consistent with increased uric acid release without changes in AMPD1/3, NT5C1A, and XOR expression in the EDL muscles.

On the other hand, FoxO-mediated transcriptional upregulation of purine nucleotide-degrading enzymes could have increased purine nucleotide degradation without increases in AMP/GMP/IMP levels. This possibility is supported by our findings of increased AMPD3 protein expression in TA muscles after fasting and DEX treatment, and increased AMPD3 protein expression in myotubes after DEX treatment and caFoxO3 expression, and our previous reports showing AMPD3 overexpression is sufficient to increase adenine nucleotide degradation in TA muscles and myotubes (23, 24).

The ability of DEX and caFoxO3 to increase AMPD3 protein expression likely stems from their activation of the AMPD3 promoter region and its subsequent gene transcription, which we demonstrated to depend on the presence of FoxO binding sites. This agrees with previous studies indicating the glucocorticoid receptor (47) and FoxO transcription factors (29) as regulators of *AMPD3* gene expression. In humans, oral dosing of DEX caused an upregulation of *AMPD3* mRNA in skeletal muscle (48). In C2C12 myotubes, Kuo et al. identified *AMPD3* as one of 147 genes containing glucocorticoid binding regions in close proximity to their genomic sequence that was also upregulated by DEX treatment (47). Transgenic mice with a muscle-specific knockout of all FoxO isoforms have attenuated *AMPD3* mRNA upregulation in response to fasting- and denervation-induced atrophy (29). Given that AMPD3 expression is controlled by glucocorticoids and FoxO, which are required for muscle atrophy in response to a diverse set of upstream etiologies (31), our findings suggest that an AMPD3-mediated increase of muscle purine nucleotide degradation is a common feature of muscle atrophy.

We found that women with below-average physical performance, but equal levels of lean body mass, had higher serum uric acid (UA) and a trend for increased serum 3-MH/1-MH, suggesting that these women have accelerated myofibrillar protein turnover. This agrees with a published report showing hyperuricemia is associated with poor muscle strength in Japanese men (49) and mimics our animal data showing a positive correlation between EDL 3-MH and UA release without muscle loss in DEX-treated mice. Therefore, serum UA levels may be a sensitive biomarker for accelerated muscle protein degradation. Since reductions in contractile function and increases in protein degradation often precede loss of skeletal muscle mass and sarcopenia (25, 37, 38), our findings also suggest that serum UA could be of clinical use for predicting risk of muscle atrophy. However, it is important to note that our measures of muscle function are not able to discern whether the weakness/poor performance is due to muscle deficiencies, neural changes, or some combination of both. Longitudinal studies comparing serum UA, muscle mass, neuromuscular function, and direct measures of muscle protein degradation rates during atrophic and nonatrophic conditions would further elucidate the utility of UA as a predictor or diagnostic biomarker for sarcopenia.

The results from this study lend insight into targeting XOR as a therapy. Treatment with XOR inhibitors, such as allopurinol and febuxostat, has been shown to have numerous therapeutic effects in humans and mice, such as improved endothelial function and reduced cardiovascular disease–related incidents (50), chronic kidney disease progression (51), cancer cachexia and mortality (52), and disuse-mediated muscle atrophy (12–14). Mechanistically, these effects often are attributed to preventing oxidative stress caused by XOR-produced superoxide anion. Our results suggest that muscle purine nucleotide degradation could be an upstream event causing increased XOR activity and various tissue pathologies. Indeed, loss of ATP may be a cause of dysfunction in skeletal muscle in numerous diseases (53). Further, this may explain why the loss of muscle strength, even if not corrected for muscle mass, is a prognostic factor in all-cause mortality (54). Another consideration is that systemic allopurinol treatment, while generally safe, has resulted in serious side effects, especially when given together with particular other drugs (55). Therefore, there is still a need for improved therapeutics to limit XOR activity. Future studies examining the contribution of skeletal muscle purine nucleotide degradation and cell-specific XOR activity on peripheral tissue pathologies may lead to developing novel therapeutic strategies.

In conclusion, skeletal muscle purine nucleotide degradation and purine release are increased in skeletal muscles with poor contractile function and increased protein degradation. Mechanistically, glucocorticoids and FoxO3 increase muscle purine nucleotide degradation by transcriptionally upregulating AMPD3 expression, which increases muscle purine release and drives downstream XOR activity and UA production by XOR-expressing cells. As such, preventing muscle purine nucleotide degradation could be a novel strategy for treating various pathologies associated with increased XOR activity and hyperuricemia.

## Methods

### Animals.

C57BL/6J mice (13–15 weeks) from Jackson Laboratory were housed 2–5 mice per cage with free access to food and water. For fasting studies, female mice were placed in a clean cage at 10 am with access to water and food, or water only, for 48 hours. For DEX studies, male and female mice were subcutaneously injected once daily for 5 days with 5 mg/kg body weight veterinary grade DEX (Dexium, Bimeda) or vehicle.

### Ex vivo skeletal muscle incubations.

Mice were anesthetized by i.p. injection of ketamine (90 mg/kg)/xylazine (10 mg/kg), kept on a 37°C heating pad, and provided supplemental O_2_ (100%) throughout muscle collection. Proximal and distal tendons of the EDL muscle were carefully tied with silk suture. Muscles were quickly excised, weighed, and vertically hung near resting length in 2 mL Krebs-Henseleit buffer (25 mM NaHCO_3_, 118 mM NaCl, 4.7 mM KCl, 1.2 mM MgSO_4_, 1.2 mM KH_2_PO_4_, 1.2 mM CaCl_2_, 5 mM glucose, 0.15 mM sodium pyruvate), which was continuously gassed with 95% O_2_/5% CO_2_ at 37°C. After 30 minutes’ preincubation, muscles were placed in fresh bath. Buffer samples were collected 1 and 2 hours later. At 2 hours, muscles were blotted dry and frozen in liquid nitrogen.

### Cell culture.

All cell lines were grown on 6-well plates at 37°C. C2C12 myoblasts (ATCC CRL-1772) were grown and differentiated as we have done previously (23, 24, 56). When required, adenoviruses encoding caFoxO3 or GFP were added after 4 days of differentiation, and myotubes were examined 48 hours later. BAOECs (MilliporeSigma B304-05) were grown in BAOEC growth medium (Cell Applications). AML-12 hepatocytes (ATCC CRL-2254) were grown in DMEM-F12 medium supplemented with 10% fetal bovine serum (FBS), penicillin/streptomycin (pen/strep), 10 μg/mL insulin, 5.5 μg/mL transferrin, 5 ng/mL selenium, and 40 ng/mL DEX. 3T3-L1 mouse embryonic fibroblasts (ATCC CL-173) were grown in “preadipocyte” medium (DMEM supplemented with 10% FBS and pen/strep), which was refreshed every 48 hours. After reaching 100% confluence, cells were maintained in preadipocyte medium for another 48 hours, then given “differentiation medium” (DMEM supplemented with 10% FBS, pen/strep, 1 μM DEX, 0.5 mM methylisobutylxanthine, and 1 μg/mL insulin). Cells were incubated in differentiation medium for 48 hours and then switched to “adipocyte maintenance” medium (DMEM supplemented with 10% FBS, pen/strep, 1 μg/mL insulin). Cells were maintained in adipocyte maintenance medium for 14 days (media refreshed every 48 hours). DEX (MP Biomedicals 190040) was dissolved in DMSO to a stock concentration of 100 mM. Stock solution was diluted in culture media, and DMSO volumes equal to highest experimental concentration were given for vehicle control. For exogenous purine treatment, hypoxanthine, xanthine, and allopurinol were dissolved in 1 M NaOH. Stock solutions were diluted in appropriate media for each cell type.

### Purine quantification.

Media samples were diluted 1:4 with cold (–20°C) 80% methanol with subsequent centrifugation at 15,000*g* for 10 minutes at 10°C to remove proteins. The supernatant was lyophilized using a Vacufuge plus (Eppendorf) and resuspended in H_2_O before UPLC analysis with Waters Acquity UPLC H-Class Bio system, as we have done previously (57).

### Amino acid quantification.

Amino acids were measured after derivatization of amine groups using the AccQ-Tag Ultra kit (Waters 186003836). Briefly, proteins were precipitated with methanol, and the supernatant was lyophilized using a Vacufuge plus. Samples were reconstituted in water and derivatized by combining 70 μL borate buffer, 15 μL sample, and 15 μL AccQ-Tag Ultra reagent. The samples were analyzed with an AccQ-Tag Ultra C18, 1.7 μm column (Waters 186003837) on a Waters Acquity UPLC system and quantified by absorbance at 260 nm.

### Protein degradation rates.

We adapted our previous protein degradation assay using ^3^H-labeled proteins (56, 58) to use stable isotope labeling. C2C12 myoblasts were differentiated in DMEM supplemented with 2% horse serum and ^13^C_9_^15^N-phenylalanine (130 mg/L, MilliporeSigma 608017). Four days later, myotubes were washed twice with unlabeled DMEM, with additional washes repeated 1 and 2 hours later to allow degradation of short-lived proteins. After the second chase period, atrophy treatments were initiated, and media samples were collected over time for measurement of ^13^C_9_^15^N-phenylalanine by UPLC and mass detection (Waters Acquity UPLC system with Acquity QDa mass detector).

### AMPD3 promoter luciferase reporter assay.

The 1.1 kb genomic promoter sequence ranging from 1,000 bases 5′ to 100 bases 3′ of the mouse AMPD3 transcriptional start site (TSS) was synthesized by GenScript and inserted in the XhoI/HindIII cloning site of the pNL1.1[Nluc] reporter plasmid (Promega). A consensus FoxO binding site, GTAAACAACTG, is located at 124 bases upstream of the TSS. To inactivate this site, a Q5 Site-directed Mutagenesis Kit (New England Biolabs) was used to mutate the original sequence to GTCCACAACTG. This is termed the ΔFoxO mutant. The accuracy of the wild-type and ΔFoxO promoter sequences was confirmed by Sanger sequencing (Azenta Life Sciences). Myoblasts were transfected 24 hours prior to differentiation. Nano-Glo luciferase activity reporter assays (Promega, N1630) were conducted according to the manufacturer’s protocol. Briefly, myotubes were harvested in passive lysis buffer supplied in the assay kit, and luciferase activity was measured by a plate reader. Protein concentration of the lysate was quantified by BCA assay (Pierce, Thermo Fisher Scientific). Luminescence values were normalized to total protein.

### Coculture experiments.

C2C12 myoblasts and myotubes were grown in DMEM supplemented with ^13^C_2_^15^N-Glycine (30 mg/L, Cambridge Isotope Laboratories CAS211057-02-2) to label intracellular purine nucleotides. BAOECs were grown on cell culture inserts (0.4 μM pore, ThinCert, Greiner Bio-One). At the start of treatments, BAOEC cultures were inserted into C2C12 myotube–containing wells. Media samples were collected and analyzed by UPLC/MS.

### Western blotting.

Proteins were extracted and analyzed by SDS-PAGE precisely as we have done previously (23). Equal protein loading and transfer were confirmed by capturing protein fluorescence after photoactivation of Bio-Rad stain-free gels. Primary antibodies from Invitrogen (AMPD3 PA5-76912, NT%C1A PA5-101545), Abcam (XOR ab109235), and Santa Cruz Biotechnology (AMPD1 D-7) were diluted 1:1,000 in TBS-Tween (TBS-T) plus 5% BSA. Secondary antibodies conjugated to horseradish peroxidase (Cell Signaling Technology 7074, Thermo Fisher Scientific 31444) were diluted 1:5,000 in TBS-T plus 2% BSA. Proteins were detected with Western Chemiluminescence HRP Substrate (MilliporeSigma). Band intensities were captured using a Bio-Rad ChemiDoc XRS imager and analyzed using Image Lab Software 6.1. Approximate molecular weights of protein were calculated relative to PageRuler Plus protein ladder (Thermo Fisher Scientific).

### MyHC/DAPI staining, imaging, quantification.

Myotube area was determined by quantifying the area of MyHC as we have done previously (58). MyHC was detected by immunofluorescence staining (primary antibody: A4.1025, Developmental Studies Hybridoma Bank at University of Iowa; secondary antibody: Alexa Fluor 546 goat anti-mouse IgG A11030, Life Technologies), and nuclei were stained using DAPI. MyHC area was calculated by quantifying the area of red fluorescent pixels per image using ImageJ software (NIH).

### Muscle cross section immunofluorescence.

TA muscles were excised, embedded in OCT compound (23-730-571, Thermo Fisher Scientific), and frozen in liquid nitrogen–cooled 2-methylbutane. A cryostat (Leica CM 1950) was used to obtain 10 μm muscle sections onto microslides (48311-703, VWR). The slides were fixed in 4% paraformaldehyde for 5 minutes, permeabilized with 0.1% Triton X-100/1× PBS, and blocked in 0.5% BSA/10% goat serum/1× PBS for 20 minutes. The slides were incubated overnight with anti–xanthine oxidase antibody (1:100, rabbit monoclonal, ab109235, Abcam) and anti-CD31 antibody (1:100, mouse monoclonal, 66065-2-lg, Proteintech) in 0.5% BSA/2% goat serum/1× PBS. For the secondary antibodies, the slides were washed and incubated with goat anti-rabbit IgG (H+L) Alexa Fluor 647 and goat anti-mouse Alexa Fluor 568 (1:300, A-21244 and A-11004, Thermo Fisher Scientific) in the dark for 1 hour. The slides were washed and incubated with DAPI/1× PBS for 15 minutes to detect nuclei. Slides were mounted with SlowFade Diamond (S36967, Thermo Fisher Scientific) and imaged using a Keyence BZ-X910 at 20× original magnification. Image processing was done using ImageJ.

### Human participants.

Deidentified physical function records and nonfasted blood samples were procured through the Musculoskeletal Function, Imaging, and Tissue Resource Core (FIT Core) at the Indiana Center for Musculoskeletal Health (ICMH) and stored at the Center for Musculoskeletal Health Repository. The participants’ data were classified according to physical performance into low or average groups using normative data collected by the FIT Core (59). Forty records, all female, were selected to meet the quotas for each performance group yet matched for age, height, weight, and BMI. Patients < 18 years old, > 80 years old, with a known history of kidney disease, with gout, or taking UA-lowering medications (e.g., allopurinol, febuxostat) were excluded. Two women did not undergo a dual energy x-ray absorptiometry scan and were excluded from the analysis of appendicular lean mass/height^2^.

### Statistics.

Unless stated otherwise, all data are presented as mean ± standard deviation. *P* < 0.05 was considered statistically significant. Statistical analyses were performed using GraphPad Prism 9. Data were tested for normality using the Shapiro-Wilk normality test and Spearman’s test for heteroscedasticity. Data with unequal variance were log-transformed prior to analysis. The statistical test used for each experiment is specified in each figure legend. All cell culture experiments were repeated at least twice to confirm reproducibility.

### Study approval.

All studies using animals were approved by the Indiana University School of Medicine Institutional Animal Care and Use Committee. The FIT Core has institutional review board approval from Indiana University to test all comers who provide written informed consent. Therefore, human studies have been performed in accordance with the ethical standards laid down in the 1964 Declaration of Helsinki and its later amendments.

### Data availability.

Values for all individual data points are reported in the Supporting Data Values file. All relevant data are available from the corresponding author upon request.

## Author contributions

SGM designed the experiments, performed the experiments, analyzed the data, and wrote the manuscript. CM, PSH, and ASL performed experiments and analyzed data. CAW contributed reagents. JJB designed experiments and wrote the manuscript. All authors contributed to the writing and approved of the final version.

## Supplementary Material

Supplemental data

Supporting data values

## Figures and Tables

**Table 1:**
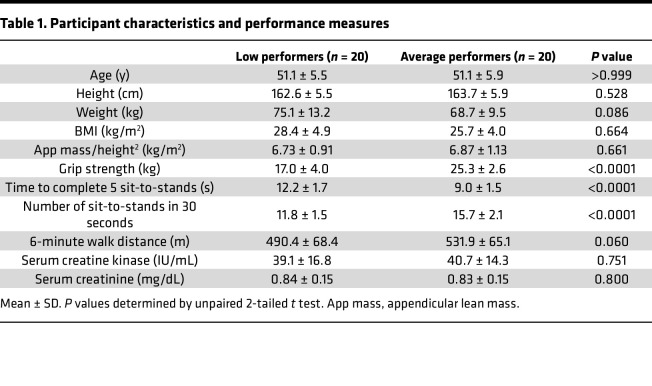
Participant characteristics and performance measures

## Version 1 11/30/2023

In-Press Preview

## Version 2 01/23/2024

Electronic publication
