# Supplementary material for: Uric acid formation is driven by crosstalk between skeletal muscle and other cell types
Source: JCI Insight. 2024 Jan 23;9(2):e171815. doi: 10.1172/jci.insight.171815 (PMC10906236; doi:10.1172/jci.insight.171815)
Supplement: Supplemental data [file jciinsight-9-171815-s062.pdf]

SUPPLEMENTAL FIGURES

Uric acid formation is driven by crosstalk between skeletal muscle and other cell types

Spencer G. Miller[1,2,3], Catalina Matias [1,2], Paul S. Hafen[1,2], Andrew S. Law[1,2], Carol A. Witczak[1,2], Jeffrey J. Brault[1,2,*]

[1]Indiana Center for Musculoskeletal Health, Indiana University School of Medicine, Indianapolis, IN, USA
[2]Department of Anatomy, Cell Biology & Physiology, Indiana University School of Medicine, Indianapolis, IN, USA
[3]Department of Kinesiology, East Carolina University, Greenville, NC, USA

***Correspondence:**
Jeffrey J. Brault, PhD
Dept. of Anatomy, Cell Biology & Physiology
Indiana University School of Medicine
635 Barnhill Dr., MS 5035
Indianapolis, IN 46202
USA
Email: jebrault@iu.edu
Phone: 1-317-278-2623

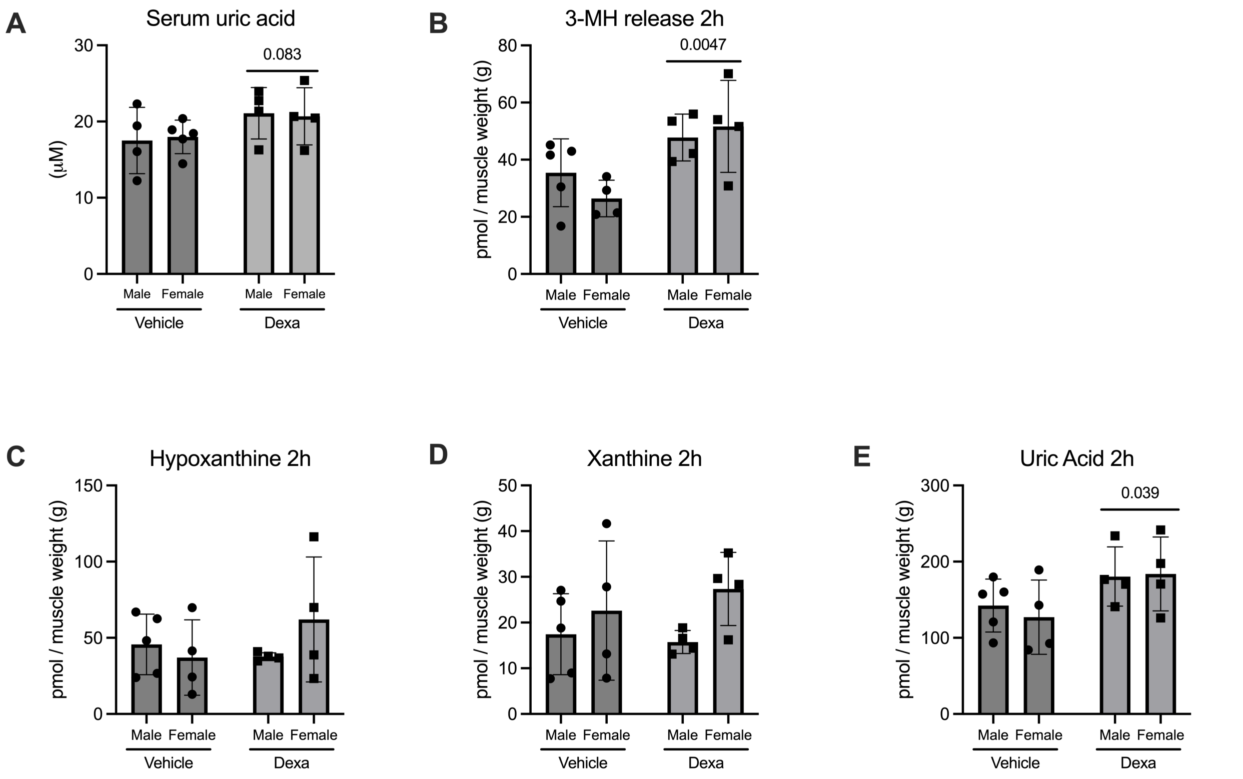

Supplemental Figure 1. Sex does not influence serum uric acid levels or uric acid release from muscles of glucocorticoid treated mice. Male and female C57BL/6J mice were treated with dexamethasone (Dexa; 5mg/kg) or vehicle for 5 days. (A) Serum was tested for uric acid. EDL muscles were incubated for 2h and media tested for (B) 3-methylhistidine, (C) hypoxanthine, (D) xanthine, and (E) uric acid. Two-way ANOVA: sex by treatment. No main effects of sex. P values in panels are main effects of Dexa

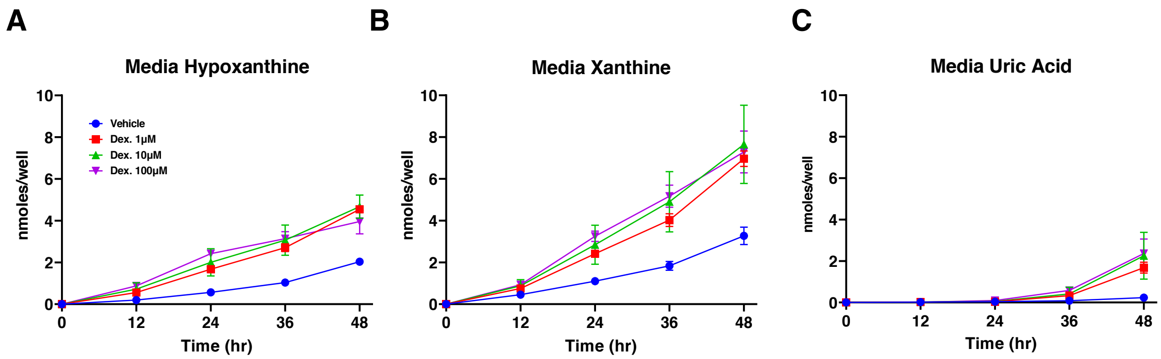

Supplemental Figure 2. C2C12 media accumulation of the purine nucleotide breakdown products hypoxanthine (A), xanthine (B) and uric acid (C) during 48h treatment with vehicle, 1, 10, or 100 μm dexamethasone (DEX).

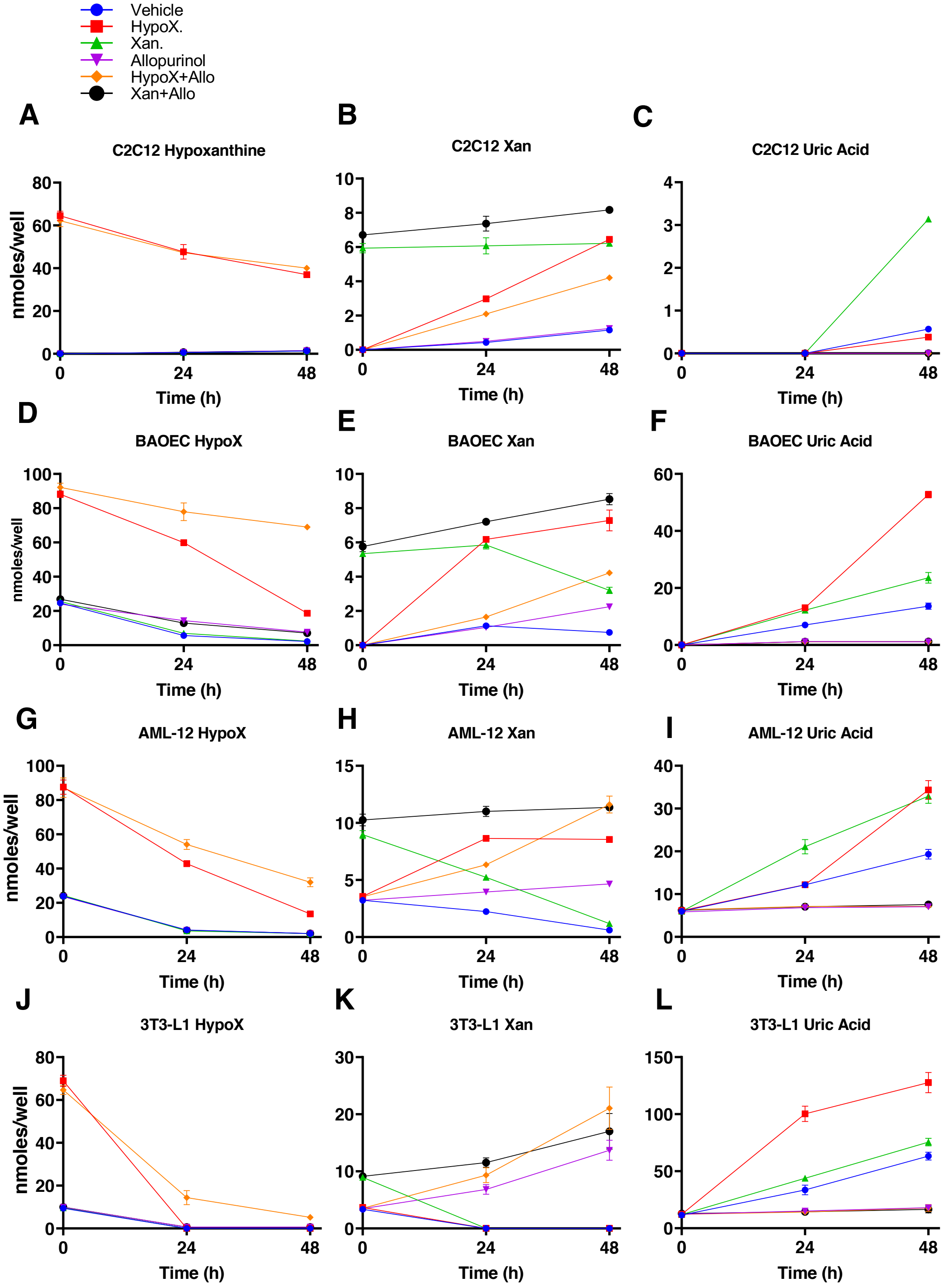

Supplemental Figure 3. Media accumulation of hypoxanthine, xanthine, and uric acid in cultured myotubes (C2C12), bovine aortic endothelial cells (BAOEC), adipocytes (3T3-L1), and hepatocytes (AML-12). Cells were treated for 48 h with vehicle, 50 μM hypoxanthine, 10 μM xanthine, ± 100 μM allopurinol.
